# Long non‐coding RNA LINC00467 drives hepatocellular carcinoma progression via inhibiting NR4A3

**DOI:** 10.1111/jcmm.14942

**Published:** 2020-03-03

**Authors:** Haihao Wang, Qiannan Guo, Kan‐Paatib Barnabo Nampoukime, Peiwen Yang, Ke Ma

**Affiliations:** ^1^ Division of Cardiothoracic and Vascular Surgery Tongji Hospital Huazhong University of Science and Technology Wuhan China; ^2^ Reproductive Medicine Center Tongji Hospital Huazhong University of Science and Technology Wuhan China; ^3^ Division of Infectious Disease Tongji Hospital Huazhong University of Science and Technology Wuhan China

**Keywords:** hepatocellular carcinoma, LINC00467, long non‐coding RNA, NR4A3, oncogene

## Abstract

Hepatocellular carcinoma (HCC) is a main cause of cancer‐related deaths globally. Long non‐coding RNAs (lncRNAs) play important roles in diverse cancers. Our previous microarray‐based lncRNA profiling showed that LINC00467 was highly expressed in HCC. Here, we further explored the expression, role and functional mechanism of lncRNA LINC00467 in HCC. Our findings revealed that LINC00467 was up‐regulated in HCC tissues and HCC cell lines. Increased expression of LINC00467 was positively associated with tumour size and vascular invasion. In vitro functional experiments revealed that LINC00467 accelerated HCC cell proliferation, cell cycle progression and migration and reduced HCC cell apoptosis. In vivo functional assays revealed that LINC00467 drove HCC xenograft growth and HCC cell proliferation and repressed HCC cell apoptosis in vivo. Moreover, LINC00467 inhibited NR4A3 post‐transcriptionally via interacting with NR4A3 mRNA to form double‐stranded RNA, which was further degraded by Dicer. The expression of NR4A3 was inversely associated with LINC00467 in HCC tissues. Functional rescue assays found that restore of NR4A3 expression blocked the oncogenic roles of LINC00467 in HCC. Taken together, our results demonstrated that lncRNA LINC00467 was a novel highly expressed and oncogenic lncRNA in HCC via inhibiting NR4A3. Targeting LINC00467 or enhancing NR4A3 may be potential therapeutic strategies against HCC.

## 1 INTRODUCTION

As the predominant primary liver cancer, hepatocellular carcinoma (HCC) is the sixth most common cancer in terms of incident cases and the fourth most deadly cancer in the world.^1^, ^2^, ^3^ Despite great progressions in surgical resection, tumour ablation and transarterial chemoembolization, the prognoses of most HCC patients are still dismal.^1^, ^4^ Therefore, it is extremely critical to explore novel therapeutic strategies for HCC.^5^, ^6^, ^7^ Unfortunately, until now, the molecular mechanisms driving HCC pathogenesis are still largely unclear.^8^, ^9^ Further understood of the critical mechanisms would provide potential therapeutic strategies for HCC.^10^

Recently, genome and transcriptome sequencings identified that most of human genome transcribes non‐coding RNAs, and whereas only 2% of human genome encode protein.^11^ Among these non‐coding transcripts, long non‐coding RNAs (lncRNAs) are a type of important regulatory transcripts, which have greater than 200 nucleotides in length and no translation ability.^12^, ^13^, ^14^, ^15^ Accumulating evidences have shown that lncRNAs are involved in diverse pathophysiological processes.^16^, ^17^, ^18^ Numerous lncRNAs are revealed to be deregulated in many diseases.^19^, ^20^, ^21^ Furthermore, complex and various functions of lncRNAs in different conditions have also been discovered.^22^, ^23^, ^24^, ^25^, ^26^, ^27^

The expression and function of multiple lncRNAs in HCC have been reported, such as the oncogenic lncRNA‐ATB, CRNDE, GPC3‐AS1, GIHCG, PXN‐AS1‐L, lncCAMTA1, PVT1 and the tumour‐suppressive lncRNAs H19, PXN‐AS1‐S and others in HCC.^28^, ^29^, ^30^, ^31^, ^32^, ^33^, ^34^ Due to more than 58 000 species of various lncRNAs have been identified in human cells, the expression and function of most of these lncRNAs in human HCC are still unknown.^11^ In our previous report, lncRNA microarray screening was performed to profile the expression of lncRNAs in HCC.^29^ Among the aberrantly expressed lncRNAs, we first identified lncRNA CRNDE that was highly expressed in HCC and enhances the proliferation, migration and invasion of HCC cells via miR‐217/MAPK1 axis.^29^ We further focused on another up‐regulated lncRNA LINC00467. LINC00467 has 6 exons with 797 nucleotides in length. The gene encoding LINC00467 is located in chromosome 1q32.3. Exploring The Cancer Genome Atlas (TCGA) dataset, we noted that LINC00467 was also increased in HCC tissues compared with normal samples. The only previous report about LINC00467 is that LINC00467 enhances neuroblastoma cell survival.^35^ The expression and roles of LINC00467 in other cancers, particular HCC, are still unclear.

Here, we explored the expression of LINC00467 in more clinical HCC samples and analysed the association between LINC00467 expression and clinicopathologic features. The expression of LINC00467 was also investigated in more publicly available gene expression datasets. Functional assays were undertaken to elucidate the biological functions of LINC00467 in HCC. In addition, the mechanisms underlying the function of LINC00467 in HCC were further investigated.

## 2 MATERIALS AND METHODS

### 2.1 Clinical tissues

HCC tissues and matched adjacent non‐cancerous liver tissues were acquired from 56 HCC patients at Tongji Hospital (Wuhan, China). None of these patients received chemotherapy before surgery. Ethical consent was granted from the Institutional Ethics Committee of Tongji Hospital. All patients provided written informed consent. All tissues were stored in liquid nitrogen until use.

### 2.2 Cell culture and treatment

Normal hepatocyte QSG‐7701 and HCC cell lines HepG2, SK‐HEP‐1 and Huh7 were obtained from BeNa Culture Collection. All cells were authenticated by short tandem repeat profiling. The cells were cultured in high‐glucose DMEM medium (Invitrogen) added with 10% foetal bovine serum (FBS, Invitrogen) in a culture chamber with 5% CO_2_ at 37°C. Cells were treated with 50 µmol/L α‐amanitin (Sigma‐Aldrich) for 0‐24 hours as indicated in this article.

### 2.3 RNA isolation and quantitative reverse transcription PCR (qRT‐PCR)

Total RNA was isolated using TRIzol reagent (Invitrogen). qRT‐PCR was carried out by the THUNDERBIRD SYBR^®^ qPCR Mix (Toyobo). Primers sequences were shown as follows: for LINC00467, 5′‐CCTTCTTCCTCATCATCGTC‐3′ (forward) and 5′‐CCCAGTTTCAGTCCCTCTTG‐3′ (reverse); for NR4A3, 5′‐ATAGTCTGAAAGGGAGGAGAGGTC‐3′ (forward) and 5′‐TCTGGGTGTTGAGTCTGTTAAAGC‐3′ (reverse); for GAPDH, 5′‐AACGGATTTGGTCGTATTG‐3′ (forward) and 5′‐GGAAGATGGTGATGGGATT‐3′ (reverse). All reactions were run as follows: 94°C, 60 seconds; 94°C, 30 seconds; 60°C, 30 seconds; 72°C, 60 seconds; 40 cycles. GAPDH was employed as endogenous control.

### 2.4 Vectors construction and transfection

To construct LINC00467 overexpression vector pcDNA3.1‐LINC00467 and in vitro transcription vector pSPT19‐LINC00467, LINC00467 full‐length sequences were PCR‐amplified using the Q5^®^ Hot Start High‐Fidelity DNA Polymerase (NEB) with the primers 5′‐CCCAAGCTTGTGGCGTAGGCCGGACATTT‐3′ (sense) and 5′‐CGGGATCCAATTTCCAAACTCTTTATTATGTGG‐3′ (antisense). Next, the PCR products were inserted into the Hind III and BamH I site of pcDNA3.1 vector (Invitrogen) or pSPT19 vector (Roche). To construct LINC00467 specific shRNAs, two independent cDNA oligonucleotides inhibiting LINC00467 (sh‐LINC00467‐1 and sh‐LINC00467‐2) were synthesized by GenePharma and inserted into the shRNA expression vector pGPU6/Neo (GenePharma). The shRNAs target sites were as follows: for sh‐LINC00467‐1:5′‐GACTCATGAAACCAATCTTCA‐3′; for sh‐LINC00467‐2:5′‐GATGCTCTGTAAACCACATAA‐3′.

NR4A3 overexpression vector was obtained from FulenGen (Catalog: EX‐Z4531‐M68). NR4A3‐specific shRNAs were obtained from FulenGen (Catalog: HSH112689‐nU6). Dicer‐specific siRNAs were obtained from Invitrogen. Transfections of vectors were conducted using Lipofectamine™ 3000 (Invitrogen).

### 2.5 Stable cell lines construction

To construct LINC00467 stably overexpressed HCC cells, pcDNA3.1‐LINC00467 was transfected into SK‐HEP‐1 and Huh7 cells. Then, the cells were treated with neomycin to select LINC00467 stably overexpressed cells. For the construction of LINC00467 stably silenced HCC cells, sh‐LINC00467‐1 and sh‐LINC00467‐2 were transfected into SK‐HEP‐1 and Huh7 cells. Then, the cells were treated with neomycin to select LINC00467 stably silenced cells. To construct NR4A3 stably overexpressed HCC cells, NR4A3 overexpression vector was transfected into Huh7 cells. Then, the cells were treated with puromycin to select NR4A3 stably overexpressed cells. To construct NR4A3 stably silenced HCC cells, NR4A3‐specific shRNAs were transfected into SK‐HEP‐1 cells. Then, the cells were treated with puromycin to select NR4A3 stably silenced cells. To construct LINC00467 and NR4A3 concurrently stably overexpressed HCC cells, NR4A3 overexpression vector was transfected into LINC00467 stably overexpressed SK‐HEP‐1 cells. Then, the cells were treated with neomycin and puromycin to select LINC00467 and NR4A3 concurrently stably overexpressed cells. The overexpress and silencing efficiencies of LINC00467 were measured by qRT‐PCR. The overexpress and silencing efficiencies of NR4A3 were verified by Western blot.

### 2.6 Western blot

After being washed using PBS, indicated HCC cells were lysed with RIPA buffer (Beyotime) added with protease inhibitor PMSF (Beyotime). Protein concentrations were measured by Pierce BCA Protein Assay Kit (Pierce). Total proteins were separated using sodium dodecyl sulphate‐polyacrylamide gel electrophoresis (SDS‐PAGE) and transferred onto polyvinylidene fluoride (PVDF) membrane (Invitrogen). Next, the membranes were incubated with primary antibodies (Anti‐NR4A3, ab155535, 1:1000, Abcam; Anti‐GAPDH, ab9484, 1:5000, Abcam). Subsequently, IgG‐HRP labelled goat anti‐rabbit secondary antibody (ab205718, 1:10 000, Abcam) and IgG‐HRP labelled goat antimouse secondary antibody (ab205719, 1:10 000, Abcam) were added. The immunoreactive proteins were detected by the ECL Detection System.

### 2.7 Glo cell viability and BrdU staining assay

Cellular proliferation viability of indicated HCC cells was assessed using Glo cell viability assay and BrdU staining assay. For Glo cell viability assay, 2000 indicated HCC cells per well were seeded in 96‐well plate. Cell viability was detected by the CellTiter‐Glo® Luminescent Cell Viability Assay (Promega) after 1, 2, 3, 4 days of incubation. For BrdU staining assay, indicated HCC cells were incubated overnight. BrdU (10 μg/mL) was added to the medium, and the cells were further cultured for 1 hour. Then, the cells were fixed in 4% paraformaldehyde for 10 minutes and stained using an anti‐BrdU antibody (Biocompare). The coverslips were counterstained using DAPI, and the results were collected using a fluorescence microscope and quantified via counting ten random fields.

### 2.8 Cell cycle analysis

Indicated HCC cells were trypsinized, washed and fixed in 70% ethanol for 24 hours at 4°C. After being washed, the cells were stained with propidium iodide (Beyotime) in the presence of RNase A (Beyotime) for 30 minutes at 37°C. Next, the cell cycle distribution was detected on a flow cytometer and analysed using ModFit LT version 5.0 software.

### 2.9 Apoptotic assay

Cellular apoptosis of indicated HCC cells was evaluated using Annexin V‐propidium iodide (PI) staining and flow cytometry. After treatment with 25 ng/mL doxorubicin (Selleck) for 24 hours, the cells were harvested, washed and resuspended in staining buffer. Cell apoptosis was detected using the FITC Annexin V Apoptosis Detection Kit (BD Biosciences) on FACSCalibur (BD Biosciences).

### 2.10 Transwell assay

Cellular migratory viability of indicated HCC cells was evaluated using transwell assay. Indicated HCC cells resuspended in fresh medium added with 1% FBS were plated in Transwell upper chambers (Corning Inc). DMEM medium with 10% FBS was plated to lower chambers. After further incubation for 48 hours, the cells that migrated to the bottom side of the chambers were fixed and stained for 30 minutes. The results were collected using a microscope.

### 2.11 Murine xenograft assay

Five‐six weeks old male BALB/c nude mice were acquired from Huazhong University of Science and Technology (Wuhan, China). 3 × 10^6^ indicated HCC cells were subcutaneously inoculated into the mice. Subcutaneous xenograft growth was detected weekly using a caliper, and subcutaneous xenograft volume was calculated according to 0.5 × L × S^2^ (L, longest diameter; S, shortest diameter). At the 28th day after inoculation, the mice were sacrificed. The subcutaneous xenografts were resected and weighed. Then, the subcutaneous tumours were fixed with formalin and made into paraffin‐embedded sections. The paraffin‐embedded sections were stained with primary antibodies (Anti‐Ki67, ab15580, 1 µg/mL, Abcam; Anti‐cleaved caspase‐3, ab2302, 1:50, Abcam) following the routine immunohistochemistry (IHC) method. The Institutional Ethics Committee of Tongji Hospital (Wuhan, China) approved the murine xenograft assay.

### 2.12 Isolation of cytoplasmic and nuclear RNA

Cytoplasmic and nuclear RNA were isolated by the PARIS Kit (Invitrogen) following the provided protocol. Next, the localization of RNA was assessed by qRT‐PCR.

### 2.13 RNA pull‐down assay

LINC00467 was in vitro transcribed from the vector pSPT19‐LINC00467 and biotin‐labelled using the Biotin RNA Labelling Mix (Roche) and T7 RNA polymerase (Roche). After being treated with DNase I (Roche), the in vitro transcribed RNA was further purified using the RNeasy Mini Kit (Qiagen). Then, 3 µg of purified biotin‐labelled LINC00467 was incubated with 1 mg of whole‐cell lysates from SK‐HEP‐1 cells at 25°C for 1 hour. The complexes were enriched using streptavidin agarose beads (Invitrogen). The RNA enriched in the pull‐down material was detected by qRT‐PCR.

### 2.14 RNA immunoprecipitation (RIP) assay

pcDNA3.1 or pcDNA3.1‐LINC00467 was transfected into SK‐HEP‐1 cells. sh‐NC, sh‐LINC00467‐1 or sh‐LINC00467‐2 was transfected into Huh7 cells. 48 hours later, RIP assays were undertaken in these cells with the Magna RIP RNA‐Binding Protein Immunoprecipitation Kit (Millipore) and a primary antibody against Dicer (5 µg per reaction; ab14601, Abcam) following the provided protocol.

### 2.15 Statistical analysis

GraphPad Prism v5.0 (GraphPad Software, La Jolla, CA, USA) was used to perform all statistical analyses. All quantitative results were shown as Mean ± standard deviation (SD). For comparison, Mann‐Whitney test, Wilcoxon signed‐rank test, Pearson chi‐squared test, one‐way analysis of variance (ANOVA) followed by Dunnett's multiple comparison test, Student's *t* test, Kruskal‐Wallis test followed by Dunn's multiple comparison test and Pearson's correlation analysis were utilized as indicated in figure legends. Probability values of less than .05 were considered significantly.

## 3 RESULTS

### 3.1 LINC00467 was highly expressed in HCC

**Figure 1:**
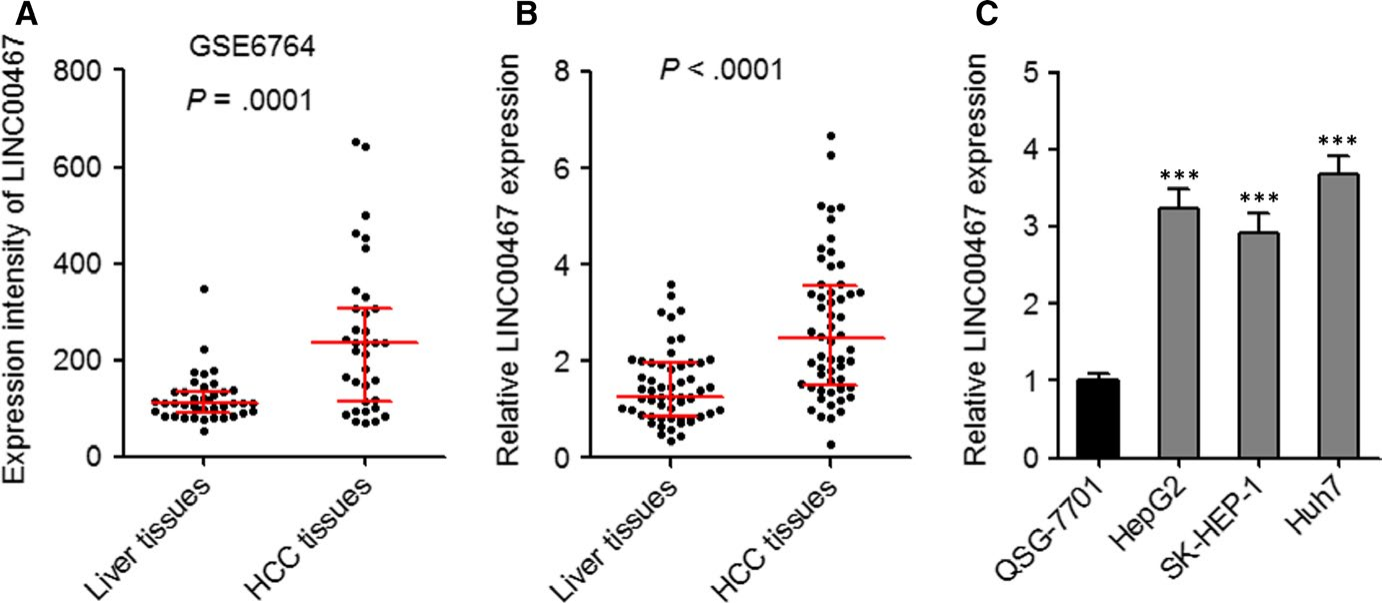
The expression pattern of LINC00467 in HCC. (A) Expression intensity of LINC00467 in http://www.ncbi.nlm.nih.gov/geo/query/acc.cgi?acc=GSE6764 dataset which includes 35 HCC tissues and 40 non‐cancerous liver tissues. *P* = .0001 by Mann‐Whitney test, compared with liver tissues group. (B) LINC00467 expression in 56 pairs of HCC tissues and adjacent non‐cancerous liver tissues was measured by qRT‐PCR *P* < .0001 by Wilcoxon signed‐rank test, compared with liver tissues group. (C) LINC00467 expression in normal hepatocyte QSG‐7701 and HCC cell lines HepG2, SK‐HEP‐1 and Huh7 was measured by qRT‐PCR. ***P < .001 by one‐way ANOVA followed by Dunnett's multiple comparison test, compared with QSG‐7701 group

We first calculated the expression intensity of LINC00467 in GEO dataset http://www.ncbi.nlm.nih.gov/geo/query/acc.cgi?acc=GSE6764 which includes 35 HCC tissues and 40 non‐cancerous liver tissues. As presented in Figure 1A, the expression intensity of LINC00467 was markedly increased in HCC tissues than that in non‐cancerous liver tissues (*P* = .0001). Next, LINC00467 expression in 56 pairs of HCC tissues and matched non‐cancerous liver tissues was assessed through qRT‐PCR. As presented in Figure 1B, LINC00467 was also overexpressed in HCC tissues compared with matched adjacent non‐cancerous liver tissues (*P* < .0001). The association between LINC00467 expression and clinicopathologic characteristic in these 56 cases of HCC patients was presented in Table 1. Pearson chi‐squared test presented that increased expression of LINC00467 was positively associated with tumour size and vascular invasion. Third, the expression of LINC00467 in normal hepatocyte QSG‐7701 and HCC cell lines HepG2, SK‐HEP‐1 and Huh7 was assessed through qRT‐PCR. The data revealed that LINC00467 was highly expressed in HCC cell lines compared with normal hepatocyte (Figure 1C). Collectively, these findings demonstrated that LINC00467 was highly expressed in HCC tissues and cells.

**Table 1:** Correlation between LINC00467 expression and clinicopathologic characteristics in 56 cases of HCC tissues

Characteristics	Number	LINC00467		*P* value^a^
		Low	High	
Age (y)				.577
≤60	36	17	19	
>60	20	11	9	
Gender				.778
Male	37	18	19	
Female	19	10	9	
HBs antigen				.703
Positive	48	23	25	
Negative	8	5	3	
Tumour size				.013
≤3 cm	35	22	13	
>3 cm	21	6	15	
Vascular invasion				.018
Yes	16	4	12	
No	40	24	16	
Tumour number				.485
Single	46	24	22	
Multiple	10	4	6	

a *P* value was acquired by Pearson chi‐squared test.

### 3.2 Overexpression of LINC00467 enhanced proliferation and migration of HCC cells

**Figure 2:**
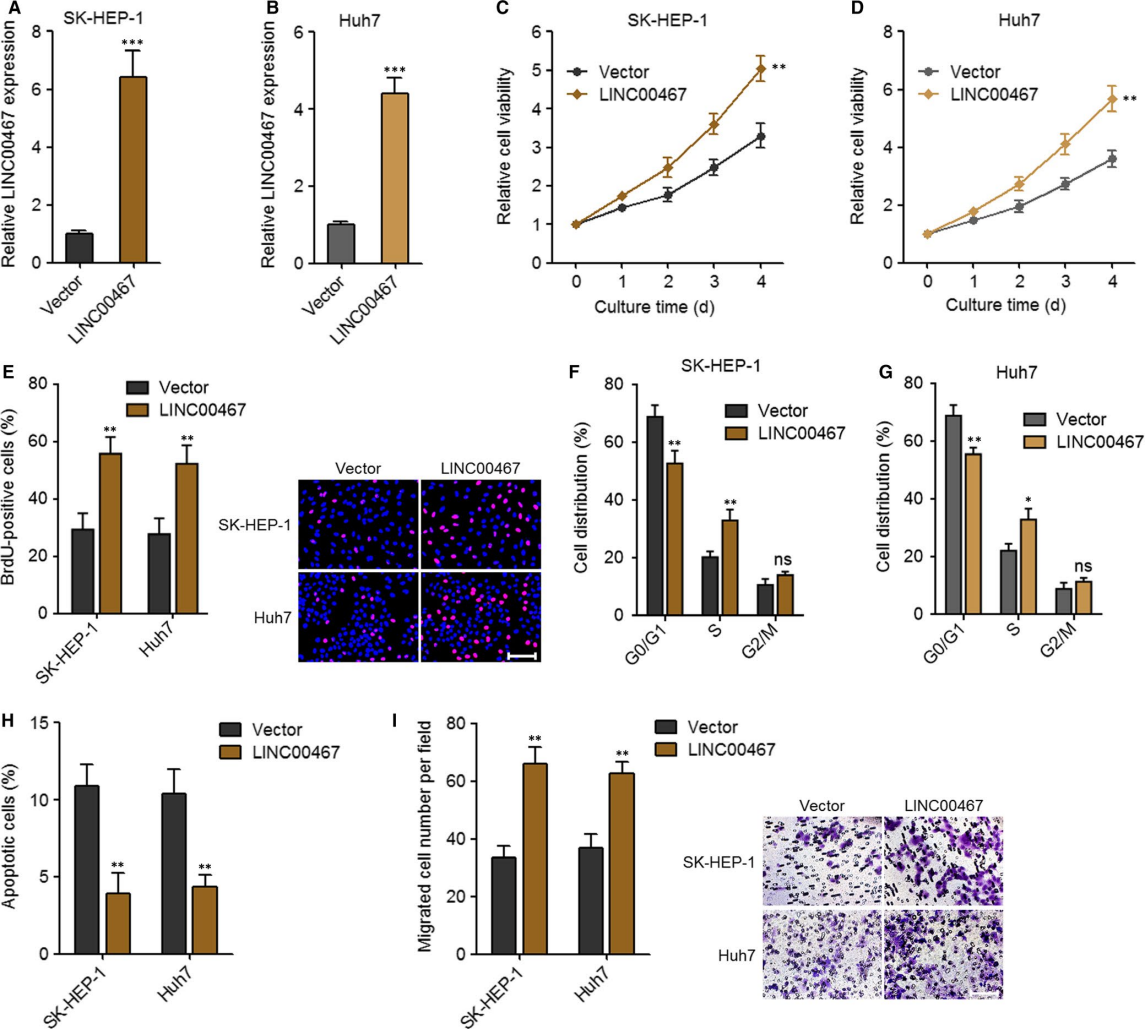
Overexpression of LINC00467 plays oncogenic roles in HCC cell proliferation, apoptosis and migration. (A) Overexpression efficiency of LINC00467 in SK‐HEP‐1 cells was verified by qRT‐PCR. (B) Overexpression efficiency of LINC00467 in Huh7 cells was verified by qRT‐PCR. (C) Glo cell viability assay showed that overexpression of LINC00467 accelerated SK‐HEP‐1 cell proliferation. (D) Glo cell viability assay showed that overexpression of LINC00467 accelerated Huh7 cell proliferation. (E) BrdU staining assay showed that overexpression of LINC00467 increased the proliferative cell number of SK‐HEP‐1 and Huh7 cells. Scale bar, 100 μm. (F) Cell cycle analysis showed the percentages of cells in each cell cycle phase after propidium iodide staining of LINC00467 overexpressed and control SK‐HEP‐1 cells. (G) Cell cycle analysis showed the percentages of cells in each cell cycle phase after propidium iodide staining of LINC00467 overexpressed and control Huh7 cells. (H) Annexin V‐PI staining and flow cytometric analyses showed that overexpression of LINC00467 reduced apoptotic cell number of SK‐HEP‐1 and Huh7 cells. (I) Transwell migration assay showed that overexpression of LINC00467 accelerated cell migration of SK‐HEP‐1 and Huh7 cells. Scale bar, 100 μm. **P < .01, ***P < .001 by Student's *t* test, compared with vector group

To evaluate the function of LINC00467 in HCC, we constructed LINC00467 stably overexpressed SK‐HEP‐1 and Huh7 cells via transfection of LINC00467 overexpression vectors (Figure 2A,B). Glo cell viability assay and BrdU staining assay were undertaken to evaluate cell proliferation ability. Glo cell viability assay revealed that cell proliferation ability was markedly accelerated by LINC00467 in both SK‐HEP‐1 and Huh7 cells (Figure 2C,D). BrdU staining assay further verified that the proliferative cell number was significantly increased by LINC00467 in both SK‐HEP‐1 and Huh7 cells (Figure 2E). Cell cycle analysis revealed that LINC00467 accelerated cell cycle progression in both SK‐HEP‐1 and Huh7 cells (Figure 2F,G). Annexin V‐PI staining and flow cytometric analyses were applied to detect cell apoptosis. The results showed that Annexin V+PI‐ apoptotic cell number was markedly reduced by LINC00467 in both SK‐HEP‐1 and Huh7 cells (Figure 2H). Transwell migration assay was applied to evaluate cell migration. Overexpression of LINC00467 significantly increased migration ability of both SK‐HEP‐1 and Huh7 cells (Figure 2I). Thus, these findings suggested that overexpression of LINC00467 promoted proliferation and cell cycle progression, repressed apoptosis and promoted migration of HCC cells.

### 3.3 Knockdown of LINC00467 decreased the proliferation and migration of HCC cells

**Figure 3:**
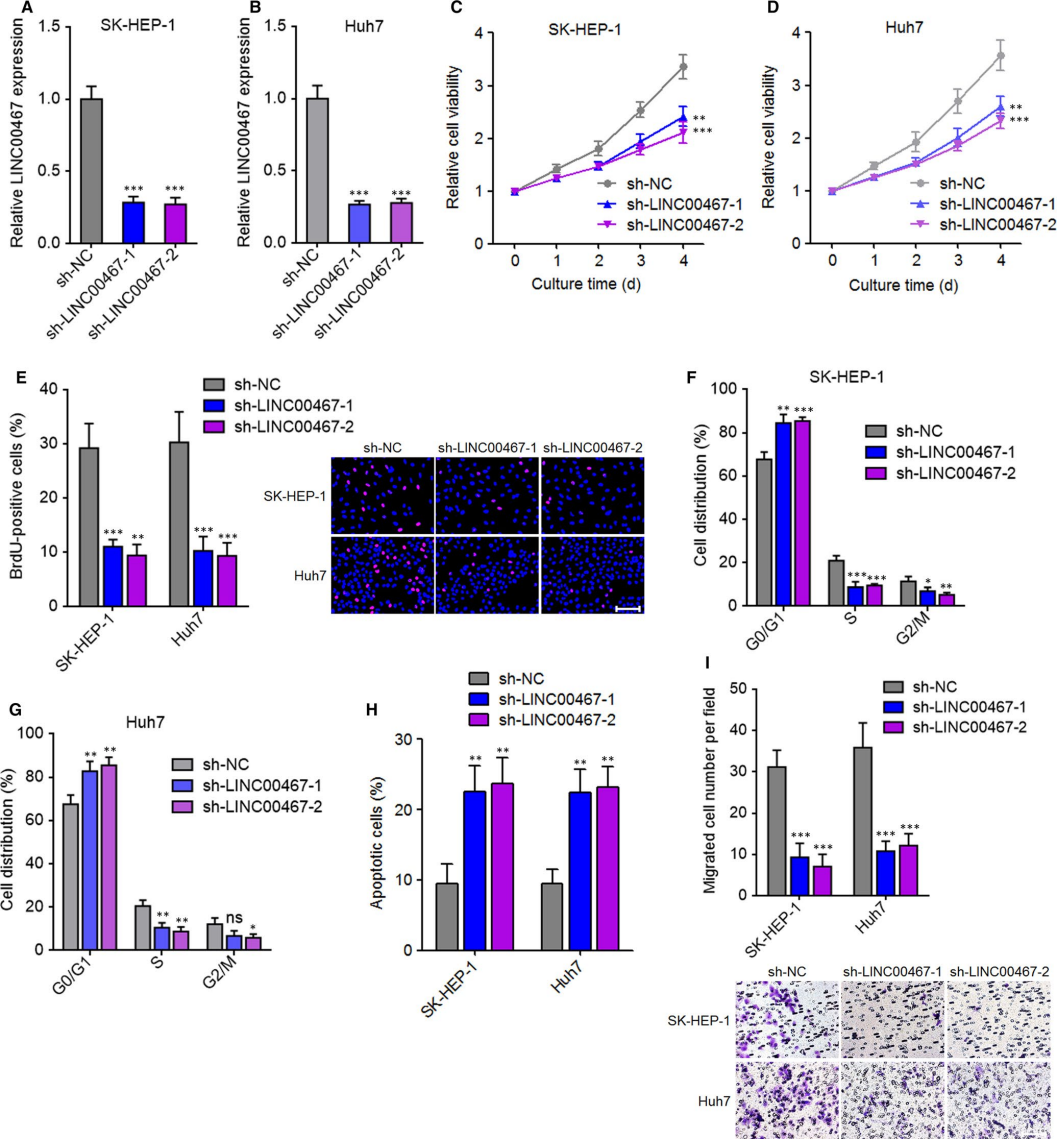
Knockdown of LINC00467 plays tumour‐suppressive roles in HCC cell proliferation, apoptosis and migration. (A) Knockdown efficiency of LINC00467 in SK‐HEP‐1 cells was verified by qRT‐PCR. (B) Knockdown efficiency of LINC00467 in Huh7 cells was verified by qRT‐PCR. (C) Glo cell viability assay showed that knockdown of LINC00467 slowed down SK‐HEP‐1 cell proliferation. (D) Glo cell viability assay showed that knockdown of LINC00467 slowed down Huh7 cell proliferation. (E) BrdU staining assay showed that knockdown of LINC00467 reduced the proliferative cell number of SK‐HEP‐1 and Huh7 cells. Scale bar, 100 μm. (F) Cell cycle analysis showed the percentages of cells in each cell cycle phase after propidium iodide staining of LINC00467 depleted and control SK‐HEP‐1 cells. (G) Cell cycle analysis showed the percentages of cells in each cell cycle phase after propidium iodide staining of LINC00467 depleted and control Huh7 cells. (H) Annexin V‐PI staining and flow cytometric analyses showed that knockdown of LINC00467 increased apoptotic cell number of SK‐HEP‐1 and Huh7 cells. (I) Transwell migration assay showed that knockdown of LINC00467 slowed down cell migration of SK‐HEP‐1 and Huh7 cells. Scale bar, 100 μm. **P < .01, ***P < .001 by one‐way ANOVA followed by Dunnett's multiple comparison test, compared with sh‐NC group

LINC00467 was stably knocked down in SK‐HEP‐1 and Huh7 cells via transfection of two independent LINC00467‐specific shRNAs (Figure 3A,B). Glo cell viability assay showed that cell proliferation ability was markedly decreased by LINC00467 knockdown in both SK‐HEP‐1 and Huh7 cells (Figure 3C,D). BrdU staining assay further verified that the proliferative cell number was markedly reduced by LINC00467 knockdown in both SK‐HEP‐1 and Huh7 cells (Figure 3E). Cell cycle analysis revealed that LINC00467 knockdown induced cell cycle arrest in both SK‐HEP‐1 and Huh7 cells (Figure 3F,G). Annexin V‐PI staining and flow cytometric analyses revealed that Annexin V+ PI‐ apoptotic cell number was significantly increased by LINC00467 knockdown in both SK‐HEP‐1 and Huh7 cells (Figure 3H). Transwell migration assay showed that LINC00467 silencing significantly reduced migration ability of both SK‐HEP‐1 and Huh7 cells (Figure 3I). Collectively, these findings demonstrated that silencing of LINC00467 suppressed proliferation, promoted apoptosis and repressed migration of HCC cells.

### 3.4 LINC00467 enhanced HCC tumour growth in vivo

**Figure 4:**
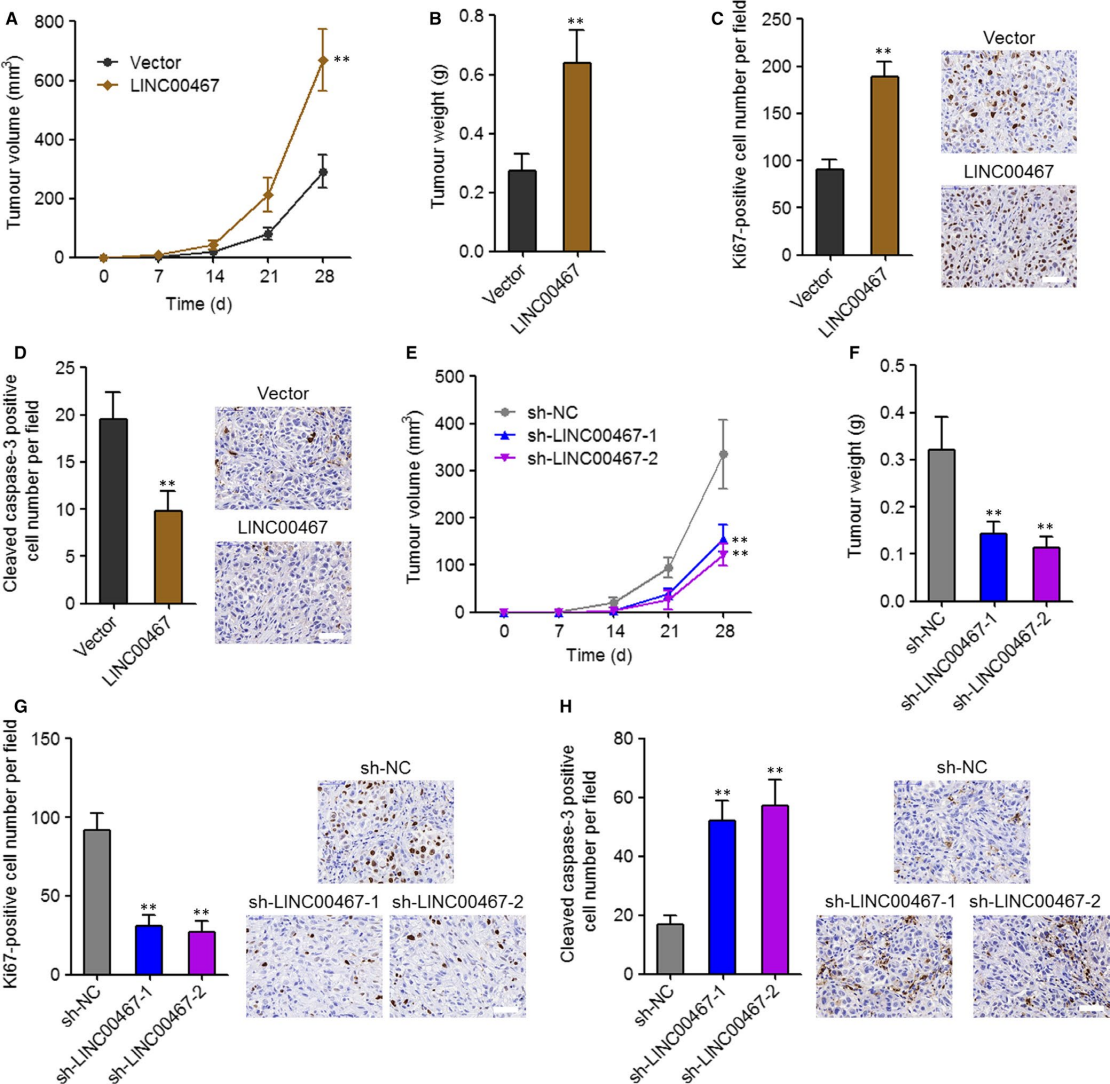
LINC00467 promoted HCC tumour growth in vivo. (A) LINC00467 stably overexpressed, and control SK‐HEP‐1 cells were subcutaneously injected into nude mice. Tumour volume was measured every 7 d. (B) Subcutaneous tumour weight was measured at the 28th day after injection. (C) Ki67 staining assay showed that overexpression of LINC00467 accelerated cell proliferation in vivo. Scale bar, 50 μm. (D) Cleaved caspase‐3 staining assay showed that overexpression of LINC00467 inhibited cell apoptosis in vivo. Scale bar, 50 μm. For A‐D, **P < .01 by Mann‐Whitney test, compared with vector group. (E) LINC00467 stably depleted, and control SK‐HEP‐1 cells were subcutaneously injected into nude mice. Tumour volume was measured every 7 days. (F) Subcutaneous tumour weight was measured at the 28th day after injection. (G) Ki67 staining assay showed that knockdown of LINC00467 inhibited cell proliferation in vivo. Scale bar, 50 μm. (H) Cleaved caspase‐3 staining assay showed that knockdown of LINC00467 promoted cell apoptosis in vivo. Scale bar, 50 μm. For E‐H, **P < .01 by Kruskal‐Wallis test followed by Dunn's multiple comparison test, compared with sh‐NC group

To elucidate the function of LINC00467 in HCC in vivo, LINC00467 stably overexpressed and control SK‐HEP‐1 cells were subcutaneously inoculated into nude mice. The subcutaneous xenograft growth was significantly increased by LINC00467 overexpression (Figure 4A,B). Proliferation marker Ki67 staining assay revealed that in vivo cell proliferation was markedly increased by LINC00467 overexpression (Figure 4C). Apoptosis marker cleaved caspase‐3 staining assay showed that in vivo cell apoptosis was significantly decreased by LINC00467 overexpression (Figure 4D). In addition, LINC00467 stably silenced and control SK‐HEP‐1 cells were also subcutaneously inoculated into nude mice. The subcutaneous xenograft growth was significantly reduced by LINC00467 knockdown (Figure 4E,F). Ki67 staining assay showed that in vivo cell proliferation was markedly decreased by LINC00467 silencing (Figure 4G). Apoptosis marker cleaved caspase‐3 staining assay showed that in vivo cell apoptosis was significantly increased by LINC00467 knockdown (Figure 4H). Taken together, these findings showed that LINC00467 promoted HCC cell proliferation, repressed HCC cell apoptosis and promoted HCC tumour growth in vivo.

### 3.5 LINC00467 repressed NR4A3 expression in post‐transcriptional level via Dicer‐dependent RNA splicing

**Figure 5:**
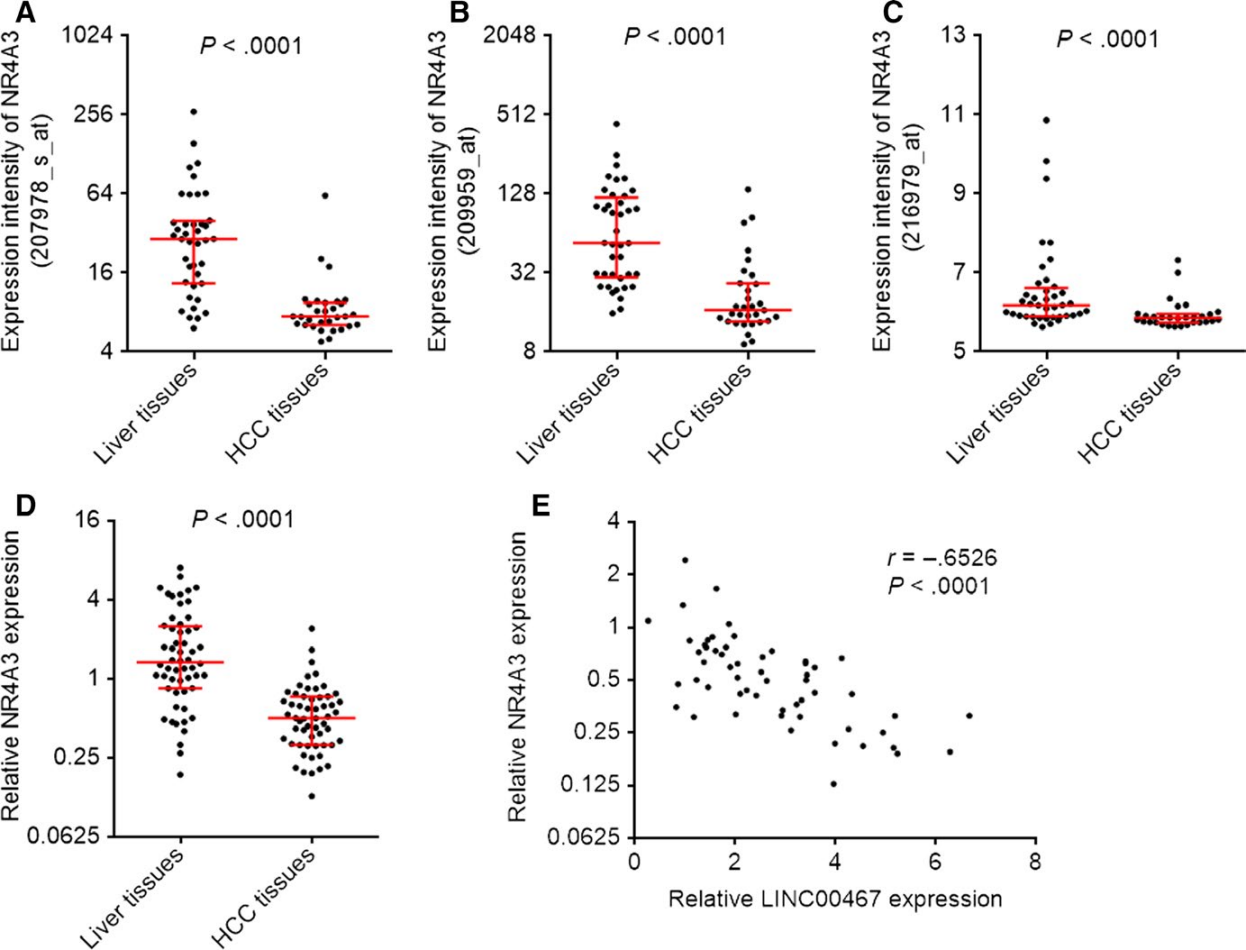
The negative correlation between LINC00467 and NR4A3 expression in HCC tissues. (A) Expression intensity of NR4A3 detected by probe 207978_s_at in http://www.ncbi.nlm.nih.gov/geo/query/acc.cgi?acc=GSE6764 dataset which includes 35 HCC tissues and 40 non‐cancerous liver tissues. *P* < .0001 by Mann‐Whitney test, compared with liver tissues group. (B) Expression intensity of NR4A3 detected by probe 209959_at in http://www.ncbi.nlm.nih.gov/geo/query/acc.cgi?acc=GSE6764 dataset. *P* < .0001 by Mann‐Whitney test, compared with liver tissues group. (C) Expression intensity of NR4A3 detected by probe 216979_at in http://www.ncbi.nlm.nih.gov/geo/query/acc.cgi?acc=GSE6764 dataset. *P* < .0001 by Mann‐Whitney test, compared with liver tissues group. (D) NR4A3 expression in 56 pairs of HCC tissues and adjacent non‐cancerous liver tissues was measured by qRT‐PCR *P* < .0001 by Wilcoxon signed‐rank test, compared with liver tissues group. (E) Pearson's correlation analysis revealed NR4A3 expression was negatively correlated with LINC00467 expression in 56 HCC tissues used in D

Atmadibrata et al^35^ previously performed mRNA microarray analysis to search the genes regulated by LINC00467 after transfection of LINC00467 siRNA into neuroblastoma cells. They identified 108 down‐regulated genes and 48 up‐regulated genes after LINC00467 knockdown in neuroblastoma cells.^35^ To search potential genes regulated by LINC00467 in HCC, we analysed the expression correlation between LINC00467 and these 108 down‐regulated plus 48 up‐regulated genes using http://www.ncbi.nlm.nih.gov/geo/query/acc.cgi?acc=GSE9843 dataset, which includes 91 HCC tissues. Furthermore, the expression correlation between LINC00467 and these 108 down‐regulated plus 48 up‐regulated genes was also analysed using http://www.ncbi.nlm.nih.gov/geo/query/acc.cgi?acc=GSE6764 dataset, which includes 75 specimens. As shown in Table S1, five down‐regulated genes after LINC00467 knockdown were positively correlated with LINC00467 in both http://www.ncbi.nlm.nih.gov/geo/query/acc.cgi?acc=GSE9843 and http://www.ncbi.nlm.nih.gov/geo/query/acc.cgi?acc=GSE6764 dataset. Seven up‐regulated genes after LINC00467 knockdown were negatively correlated with LINC00467 in both http://www.ncbi.nlm.nih.gov/geo/query/acc.cgi?acc=GSE9843 and http://www.ncbi.nlm.nih.gov/geo/query/acc.cgi?acc=GSE6764 dataset (Table S1). Among these genes, we noted NR4A3, which was up‐regulated after LINC00467 knockdown and inversely associated with LINC00467 in HCC tissues. NR4A3 is a member of nuclear receptors superfamily and frequently reported to act as a tumour suppressor in gastric, breast and lung cancer.^36^, ^37^, ^38^ Therefore, we further investigated whether LINC00467 regulates NR4A3 and whether the modulation of NR4A3 mediates the oncogenic roles of LINC00467 in HCC. Analysing http://www.ncbi.nlm.nih.gov/geo/query/acc.cgi?acc=GSE6764 dataset reveals that the expression intensities of three probes detecting NR4A3 were all markedly down‐regulated in HCC tissues than that in non‐cancerous liver tissues (*P* < .0001) (Figure 5A‐C). We further measured NR4A3 expression in the same 56 pairs of HCC tissues and matched non‐cancerous liver tissues used in Figure 1B. Reciprocal to LINC00467, NR4A3 was lowly expressed in HCC tissues compared with paired non‐cancerous liver tissues (*P* < .0001) (Figure 5D). Moreover, NR4A3 expression was negatively associated with that of LINC00467 in these 56 HCC tissues (*r* = −.6526, *P* < .0001) (Figure 5E). These data presented that NR4A3 was lowly expressed and inversely associated with LINC00467 in HCC tissues.

**Figure 6:**
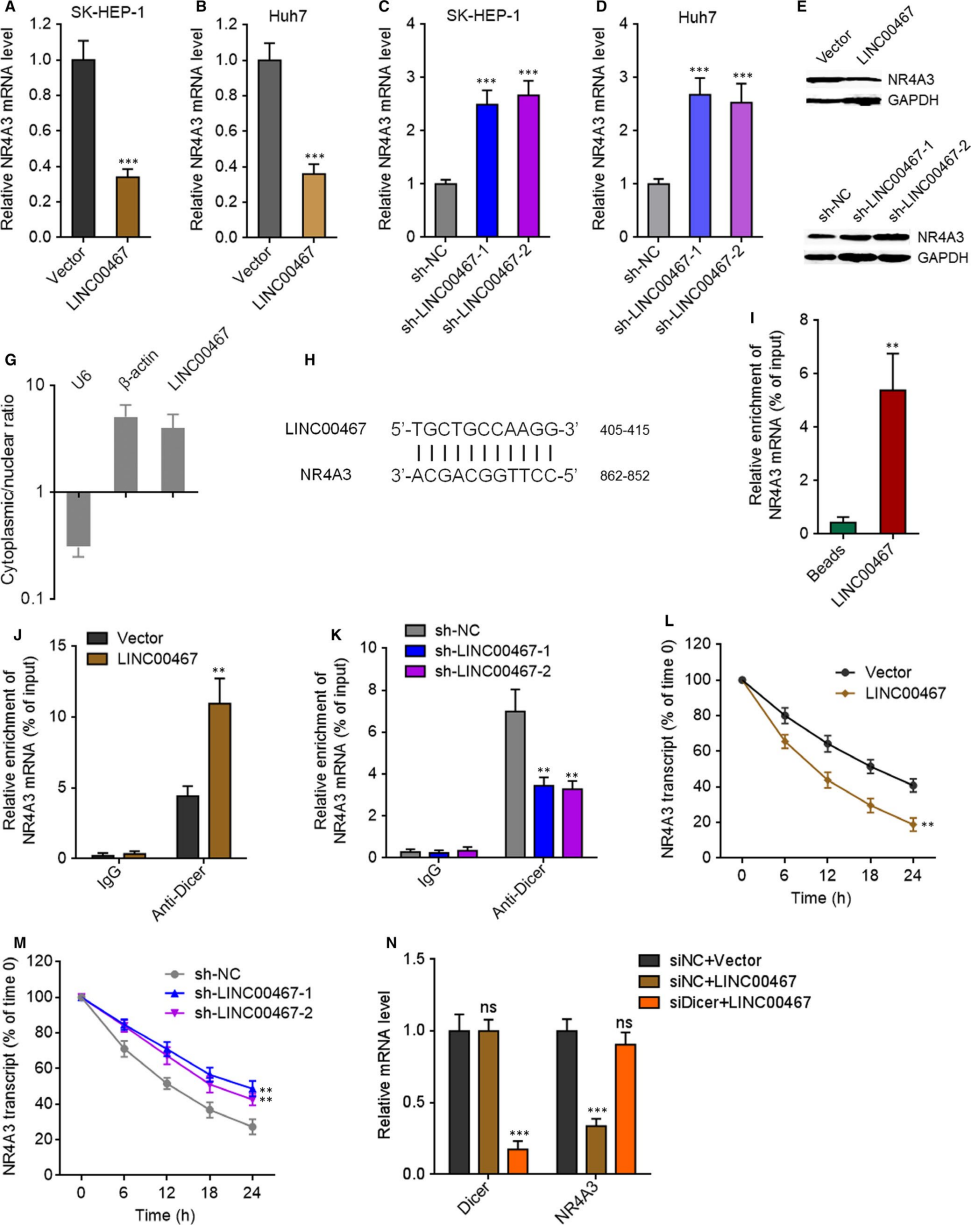
LINC00467 repressed NR4A3 expression in post‐transcriptional level via Dicer‐dependent RNA splicing. (A) NR4A3 mRNA levels in LINC00467 stably overexpressed, and control SK‐HEP‐1 cells were measured by qRT‐PCR. (B) NR4A3 mRNA levels in LINC00467 stably overexpressed, and control Huh7 cells were measured by qRT‐PCR. (C) NR4A3 mRNA levels in LINC00467 stably depleted, and control SK‐HEP‐1 cells were measured by qRT‐PCR. (D) NR4A3 mRNA levels in LINC00467 stably depleted, and control Huh7 cells were measured by qRT‐PCR. (E) NR4A3 protein levels in LINC00467 stably overexpressed, and control SK‐HEP‐1 cells were measured by Western blot. (F) NR4A3 protein levels in LINC00467 stably depleted, and control Huh7 cells were measured by Western blot. (G) Subcellular distribution of LINC00467 was determined by cytoplasmic and nuclear RNA isolation, followed by qRT‐PCR. (H) Schematic diagram of the predicted interaction region between LINC00467 and NR4A3 mRNA. (I) RNA pull‐down assays with in vitro transcribed LINC00467 was performed to investigate the interaction between LINC00467 and NR4A3 mRNA. (J) After transient transfection of LINC00467 overexpression vectors into SK‐HEP‐1 cells, RIP assays were performed using Dicer‐specific antibody, followed by qRT‐PCR to detect the enrichment of NR4A3 mRNA. (K) After transient transfection of LINC00467 shRNAs into Huh7 cells, RIP assays were performed using Dicer‐specific antibody, followed by qRT‐PCR to detect the enrichment of NR4A3 mRNA. (L) After transient transfection of LINC00467 overexpression vectors into SK‐HEP‐1 cells, the stability of NR4A3 mRNA over time was measured after blocking new RNA transcription with α‐amanitin (50 µmol/L) and normalized to 18S rRNA (a product of RNA polymerase I that is unchanged by α‐amanitin). (M) After transient transfection of LINC00467 shRNAs into Huh7 cells, the stability of NR4A3 mRNA over time was measured after blocking new RNA transcription with α‐amanitin (50 µmol/L) and normalized to 18S rRNA. (N) After transient transfection of Dicer‐specific siRNAs into LINC00467 stably overexpressed SK‐HEP‐1 cells, Dicer and NR4A3 mRNA levels were measured by qRT‐PCR. For A, B, I, J and L, **P < .01, ***P < .001 by Student's *t* test, compared with vector or beads group. For C, D, K, M and N, **P < .01, ***P < .001, ns, not significant, by one‐way ANOVA followed by Dunnett's multiple comparison test, compared with sh‐NC or siNC+vector group

Due to the negative association between the expression of LINC00467 and NR4A3 in HCC tissues, we further investigated whether LINC00467 regulates NR4A3 expression in HCC. NR4A3 mRNA and protein levels in LINC00467 stably overexpressed, and silenced HCC cells were measured through qRT‐PCR and Western blot. NR4A3 mRNA and protein levels were reduced by LINC00467 overexpression and increased by LINC00467 knockdown in HCC cells (Figure 6A‐F). Next, we investigated the detailed molecular mechanisms underlying the repression of NR4A3 by LINC00467. Subcellular distribution of LINC00467 was determined by cytoplasmic and nuclear RNA isolation and qRT‐PCR. Our data presented that LINC00467 was predominantly localized in the cytoplasm (Figure 6G). Nuclear lncRNAs mainly regulate mRNA expression transcriptionally, whereas cytoplasmic lncRNAs mainly regulate mRNA expression post‐transcriptionally. Therefore, we further investigated whether LINC00467 regulates NR4A3 mRNA stability via potential RNA‐RNA interaction. The potential interaction between LINC00467 and NR4A3 mRNA was predicted by Nucleotide Blast (https://blast.ncbi.nlm.nih.gov/Blast.cgi). Intriguingly, we identified a completely reversely complementary region between LINC00467 and coding sequences of NR4A3 mRNA (Figure 6H). To investigate whether LINC00467 binds NR4A3 mRNA via the complementary region, RNA‐RNA pull‐down assays were performed with in vitro transcribed LINC00467. Our data showed that NR4A3 transcript was enriched in LINC00467 group (Figure 6I), supporting the interaction between LINC00467 and NR4A3 mRNA. Dicer has been demonstrated to splice double‐stranded RNA (dsRNA) via binding these dsRNA.^39^ To investigate whether the dsRNA formed by LINC00467 and NR4A3 mRNA is spliced and bound by Dicer, we performed RIP assay in HCC cells. Compared with IgG group, Dicer antibody group showed significant enrichment of NR4A3 mRNA (Figure 6J,K). Furthermore, overexpression of LINC00467 markedly increased the binding of NR4A3 mRNA to Dicer, whereas knockdown of LINC00467 markedly decreased the binding of NR4A3 mRNA to Dicer (Figure 6J,K). Next, we detected the effects of LINC00467‐NR4A3 dsRNA on NR4A3 mRNA stability. After transient transfection of LINC00467 overexpression vectors or shRNAs into HCC cells, the cells were treated with α‐amanitin to inhibit novel RNA transcription. Then, the loss of NR4A3 transcript was detected. Our data showed that LINC00467 overexpression markedly shortened the half‐life of NR4A3 mRNA, whereas LINC00467 silencing markedly elongated the half‐life of NR4A3 mRNA (Figure 6L,M), supporting that LINC00467‐NR4A3 dsRNA reduced NR4A3 transcript stability and promoted NR4A3 transcript degradation. To elucidate whether the roles of LINC00467‐NR4A3 dsRNA on NR4A3 mRNA were dependent on Dicer, we silenced Dicer in LINC00467 overexpressed SK‐HEP‐1 cells. Our data presented that silencing of Dicer abrogated the down‐regulation of NR4A3 mRNA caused by LINC00467 overexpression (Figure 6N). Taken together, the data demonstrated that LINC00467 repressed NR4A3 expression in post‐transcriptional level via Dicer‐dependent RNA splicing.

### 3.6 NR4A3 mediated the oncogenic roles of LINC00467 in HCC

**Figure 7:**
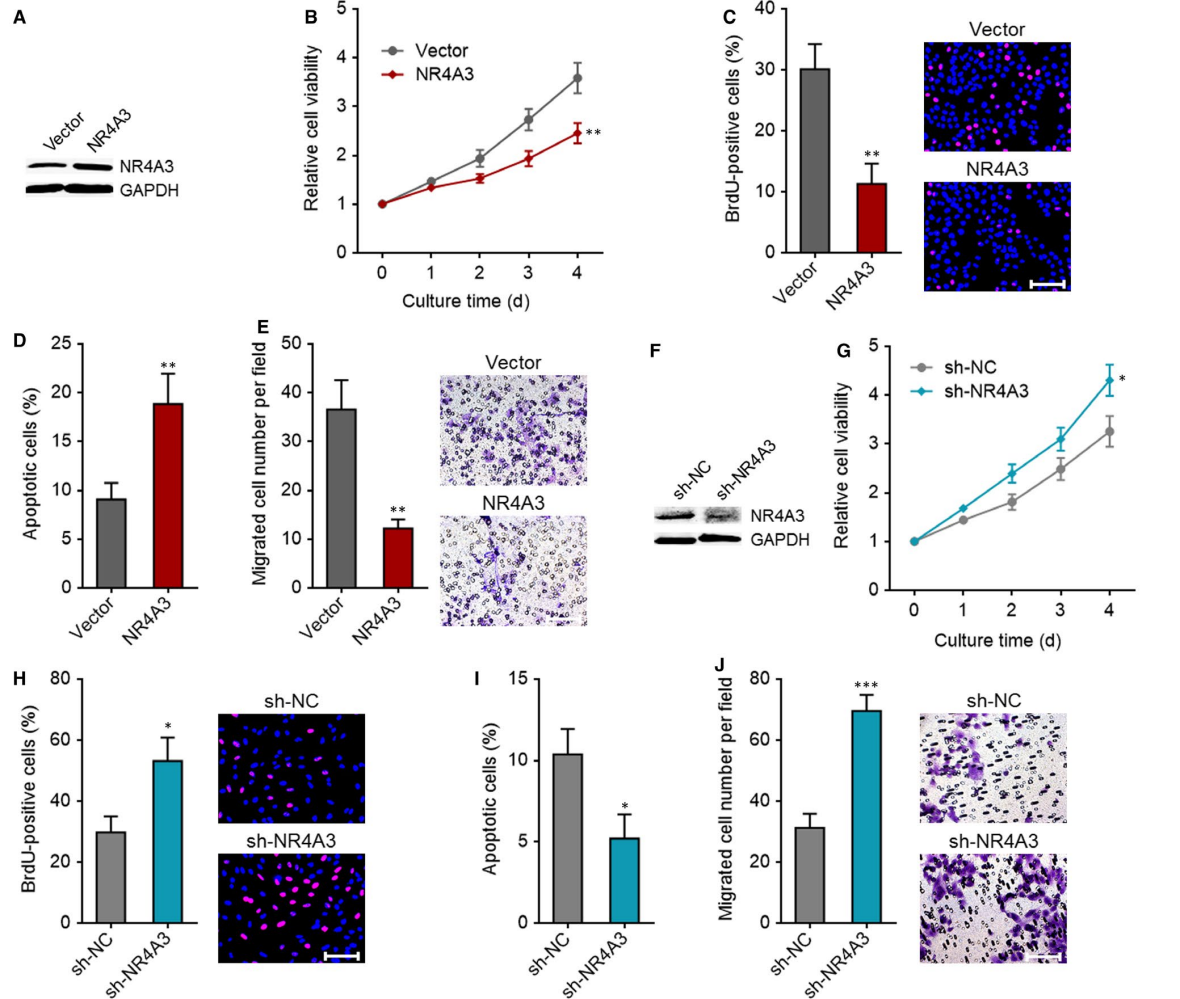
NR4A3 plays tumour‐suppressive roles in HCC cell proliferation, apoptosis and migration. (A) Overexpression efficiency of NR4A3 in Huh7 cells was verified by Western blot. (B) Glo cell viability assay showed that overexpression of NR4A3 slowed down cell proliferation. (C) BrdU staining assay showed that overexpression of NR4A3 reduced the proliferative cell number. Scale bar, 100 μm. (D) Annexin V‐PI staining and flow cytometric analyses showed that overexpression of NR4A3 increased apoptotic cell number. (E) Transwell migration assay showed that overexpression of NR4A3 repressed cell migration. Scale bar, 100 μm. (F) Knockdown efficiency of NR4A3 in SK‐HEP‐1 cells was verified by Western blot. (G) Glo cell viability assay showed that knockdown of NR4A3 accelerated cell proliferation. (H) BrdU staining assay showed that knockdown of NR4A3 increased the proliferative cell number. Scale bar, 100 μm. (I) Annexin V‐PI staining and flow cytometric analyses showed that knockdown of NR4A3 decreased apoptotic cell number. (J) Transwell migration assay showed that knockdown of NR4A3 promoted cell migration. Scale bar, 100 μm. *P < .05, **P < .01, ***P < .001 by Student's *t* test, compared with vector group or sh‐NC group

To investigate whether NR4A3 down‐regulation was a critical mediator of the roles of LINC00467 in HCC, we first confirmed the roles of NR4A3 in HCC. NR4A3 stably overexpressed Huh7 cells were constructed via transfection of NR4A3 overexpression vectors (Figure 7A). Glo cell viability assay showed that cell proliferation ability was reduced by NR4A3 overexpression (Figure 7B). BrdU staining assay further verified that the proliferative cell number was reduced by NR4A3 overexpression (Figure 7C). Annexin V‐PI staining and flow cytometric analyses revealed that Annexin V+PI‐ apoptotic cell number was increased by NR4A3 overexpression (Figure 7D). Transwell migration assay revealed that enhanced expression of NR4A3 augmented cell migration ability (Figure 7E). In addition, we stably knocked down NR4A3 in SK‐HEP‐1 cells via transfection of NR4A3‐specific shRNAs (Figure 7F). Glo cell viability assay demonstrated that cell proliferation ability was augmented by NR4A3 knockdown (Figure 7G). BrdU staining assay further verified that the proliferative cell number was increased by NR4A3 knockdown (Figure 7H). Annexin V‐PI staining and flow cytometric analyses revealed that Annexin V+PI‐ apoptotic cell number was reduced by NR4A3 knockdown (Figure 7I). Transwell migration assay showed that NR4A3 knockdown increased cell migration ability (Figure 7J). Collectively, these findings demonstrated that NR4A3 inhibited proliferation, promoted apoptosis and repressed migration of HCC cells.

**Figure 8:**
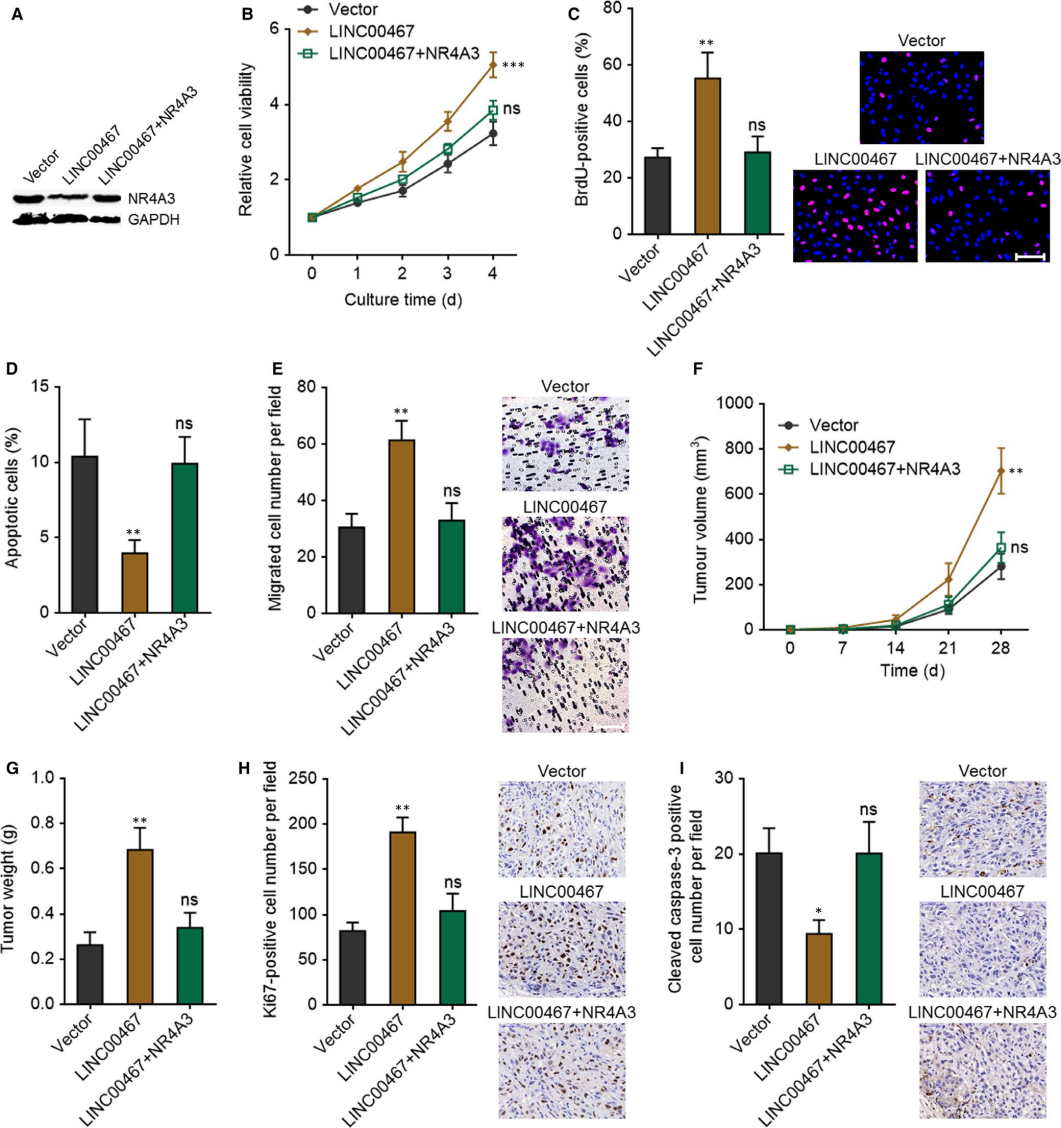
LINC00467 plays oncogenic roles in HCC cell proliferation, apoptosis and migration through NR4A3 regulation. (A) Overexpression efficiency of NR4A3 in LINC00467 stably overexpressed SK‐HEP‐1 cells was verified by Western blot. (B) Glo cell viability assay showed that overexpression of NR4A3 could largely reverse the accelerating effects of LINC00467 on cell proliferation. (C) BrdU staining assay showed that overexpression of NR4A3 could largely reverse the increasing of proliferative cell number caused by LINC00467. Scale bar, 100 μm. (D) Annexin V‐PI staining and flow cytometric analyses showed that overexpression of NR4A3 could largely reverse the reduction of apoptotic cell number caused by LINC00467. (E) Transwell migration assay showed that overexpression of NR4A3 could largely reverse the accelerating effects of LINC00467 on cell migration. Scale bar, 100 μm. For B‐E, **P < .01, ***P < .001, ns, not significant, by one‐way ANOVA followed by Dunnett's multiple comparison test, compared with vector group. (F) NR4A3 and LINC00467 concurrently stably overexpressed, and control SK‐HEP‐1 cells were subcutaneously injected into nude mice. Tumour volume was measured every 7 d. (G) Subcutaneous tumour weight was measured at the 28th day after injection. (H) Ki67 staining assay showed that overexpression of NR4A3 could largely reverse the accelerating effects of LINC00467 on in vivo cell proliferation. Scale bar, 50 μm. (I) Cleaved caspase‐3 staining assay showed that overexpression of NR4A3 could largely reverse the reduction of in vivo apoptotic cell number caused by LINC00467. Scale bar, 50 μm. For F‐I, **P < .01, ns, not significant, by Kruskal‐Wallis test followed by Dunn's multiple comparison test, compared with vector group

To investigate whether LINC00467 plays its oncogenic roles via down‐regulating NR4A3, NR4A3 was stably overexpressed in LINC00467 stably overexpressed SK‐HEP‐1 cells via transfection of NR4A3 overexpression vectors (Figure 8A). Glo cell viability assay revealed that overexpression of NR4A3 markedly attenuated the increased cell proliferation ability caused by LINC00467 overexpression (Figure 8B). BrdU staining assay further verified that overexpression of NR4A3 significantly attenuated the up‐regulation of proliferative cell number caused by LINC00467 overexpression (Figure 8C). Annexin V‐PI staining and flow cytometric analyses revealed that overexpression of NR4A3 significantly attenuated the reduction of Annexin V+ PI‐ apoptotic cell number caused by LINC00467 overexpression (Figure 8D). Transwell migration assay presented that overexpression of NR4A3 markedly attenuated the increased cell migration ability caused by LINC00467 overexpression (Figure 8E). SIPR1 and LINC00467 concurrently stably overexpressed SK‐HEP‐1 cells were subcutaneously inoculated into nude mice. Overexpression of NR4A3 significantly attenuated the increasing of subcutaneous tumour growth caused by LINC00467 overexpression (Figure 8F,G). Proliferation marker Ki67 staining assay showed that overexpression of NR4A3 significantly attenuated the increased in vivo cell proliferation caused by LINC00467 overexpression (Figure 8H). Apoptosis marker cleaved caspase‐3 staining assay presented that overexpression of NR4A3 markedly attenuated the reduced in vivo cell apoptosis caused by LINC00467 overexpression (Figure 8I). Taken together, these findings suggested that NR4A3 overexpression reversed the oncogenic roles of LINCC467 in HCC.

## 4 DISCUSSION

Great advances have been made in molecular targeted therapies in multiple cancers, such as lung cancer and melanoma.^40^, ^41^, ^42^, ^43^ Although sorafenib, lenvatinib and regorafenib have been approved by FDA for the therapy of HCC, the global survival benefits for these drugs are only about 3‐6 months.^1^ Therefore, more efficient molecule‐targeted therapies are urgently needed. Increasing studies have identified many functional lncRNAs, which play key roles in the initiation and progression of various cancers.^15^ Moreover, several clinical trials targeting RNA molecules are undergoing, which suggest that lncRNAs could be potential therapeutic targets for various diseases.^44^, ^45^ Therefore, further identifying functional lncRNAs in HCC would provide more novel candidates for HCC therapy.

In our previous microarray screening, we identified many dysregulated lncRNAs in HCC.^29^ Furthering analysing TCGA data, we focused our attention on lncRNA LINC00467. First, lncRNAs microarray results of ten pairs of fresh human HCC tissues and adjacent non‐cancerous liver tissues revealed that LINC0467 was one of the most highly expressed lncRNAs in HCC tissues compared with paired non‐cancerous liver tissues. Second, analysing the TCGA dataset revealed that LINC00467 was markedly unregulated in 374 HCC tissues compared with 50 normal liver tissues. Third, analysing the GEO dataset http://www.ncbi.nlm.nih.gov/geo/query/acc.cgi?acc=GSE6764 revealed that LINC00467 was markedly highly expressed in 35 HCC tissues compared with 40 non‐cancerous liver tissues. Therefore, we investigate the expression, roles and clinical significances of LINC00467 in HCC.

We detected LINC00467 expression in 56 pairs of HCC tissues and matched non‐cancerous liver tissues collected in our hospital and analysed the correlation between LINC00467 expression and clinicopathologic characteristic in these 56 cases of HCC. Our findings further confirmed that LINC00467 was increased in HCC tissues and positively associated with tumour size and vascular invasion. Moreover, our findings revealed that LINC00467 was also up‐regulated in HCC cell lines compared with normal hepatocyte. Therefore, these findings collectively verified that LINC00467 was highly expressed in HCC.

Further in vitro functional assays demonstrated that ectopic expression of LINC00467 markedly enhanced HCC cell proliferation and migration and repressed HCC cell apoptosis, whereas knockdown of LINC00467 significantly repressed HCC cell proliferation and migration and promoted HCC cell apoptosis. In vivo functional assays presented that overexpression of LINC00467 markedly enhanced HCC xenograft growth and HCC cell proliferation and repressed HCC cell apoptosis in vivo, whereas knockdown of LINC00467 markedly repressed HCC xenograft growth and HCC cell proliferation and promoted HCC cell apoptosis in vivo. Therefore, these findings demonstrated that LINC00467 had oncogenic roles in HCC and also suggested that targeting LINC00467 would be potential treatment strategy against HCC.

The molecular mechanisms responsible for the role of lncRNAs are diverse.^12^ Typically, lncRNAs could interact with proteins, DNAs and other RNAs and further regulate the expression of critical oncogenes or tumour suppressors in cancers.^15^, ^17^ Atmadibrata et al^35^ had performed mRNA microarray to search the genes regulated by LINC00467 knockdown in neuroblastoma cells. The correlations between the expression of LINC00467 and these LINC00467‐regulated genes were analysed in HCC tissues using GEO dataset http://www.ncbi.nlm.nih.gov/geo/query/acc.cgi?acc=GSE6764 and http://www.ncbi.nlm.nih.gov/geo/query/acc.cgi?acc=GSE9843. Among these LINC00467‐regulated genes, we noted that NR4A3, which was up‐regulated by LINC00467 knockdown, was negatively correlated with LINC00467 in both http://www.ncbi.nlm.nih.gov/geo/query/acc.cgi?acc=GSE6764 and http://www.ncbi.nlm.nih.gov/geo/query/acc.cgi?acc=GSE9843. NR4A3 (orphan nuclear receptor 4A3) belongs to the nuclear receptor superfamily. NR4A3 has been shown to regulate cell proliferation, apoptosis and migration and have generally tumour‐suppressive roles in breast cancer, lung cancer, gastric cancer and leukaemia.^36^, ^37^, ^46^ Therefore, we hypothesized that the tumour suppressor NR4A3 may mediate the oncogenic roles of LINC00467 in HCC. To validate this hypothesis, we first detected the effects of LINC00467 on NR4A3 in HCC. Our findings revealed that overexpression of LINC00467 repressed NR4A3 mRNA and protein levels in HCC cells, whereas LINC00467 silencing up‐regulated NR4A3 mRNA and protein levels in HCC cells. These findings confirmed that LINC00467 also down‐regulated NR4A3 in HCC. Mechanistically, we found that LINC00467 interacted with NR4A3 mRNA to form dsRNA, which was further degraded by Dicer. Second, functional experiments were undertaken to detect the roles of NR4A3 in HCC. Our findings revealed that overexpression of NR4A3 repressed HCC cell proliferation and migration and promoted HCC cell apoptosis, whereas NR4A3 knockdown promoted HCC cell proliferation and migration and repressed apoptosis. These data demonstrated that NR4A3 plays tumour‐suppressive role in HCC. Third, functional rescue experiments revealed that NR4A3 overexpression significantly reversed the oncogenic roles of LINC00467 in HCC. Fourth, in contrast to LINC00467, NR4A3 was down‐regulated in HCC tissues, and NR4A3 expression was inversely associated with that of LINC00467 in HCC tissues. Collectively, these findings suggested that NR4A3 was an important downstream mediator of the roles of LINC00467 in HCC.

In summary, our study suggested lncRNA LINC00467 as a novel highly expressed and oncogenic lncRNA in HCC. LINC00467 exerts its oncogenic roles via inhibiting NR4A3 post‐transcriptionally in a Dicer‐dependent manner. Targeting LINC00467 or enhancing NR4A3 may be potential therapeutic methods against HCC.

## CONFLICT OF INTEREST

None.

## AUTHOR CONTRIBUTIONS

Ke Ma and Haihao Wang designed the study. Haihao Wang, Qiannan Guo and Peiwen Yang performed the experiments. Haihao Wang, Ke Ma and Kan‐Paatib Barnabo Nampoukime analysed the data. Haihao Wang and Ke Ma wrote the manuscript.

## Data Availability

The data that support the findings of this study are available from the corresponding author upon reasonable request.
