# Supplementary material for: Long non‐coding RNA LINC00467 drives hepatocellular carcinoma progression via inhibiting NR4A3
Source: J Cell Mol Med. 2020 Mar 3;24(7):3822–36. doi: 10.1111/jcmm.14942 (PMC7171408; doi:10.1111/jcmm.14942)
Supplement: Supplementary file 1 [file JCMM-24-3822-s001.docx]

| Probe Set ID | Gene | Fold change |  | GSE9843 | |  | GSE6764 | |
| --- | --- | --- | --- | --- | --- | --- | --- | --- |
|  |  |  |  | Spearman r | P value |  | Spearman r | P value |
| 240344_x_at | LYRM7 | -1.999 |  | 0.267 | 0.0105 |  | 0.3978 | 0.0004 |
| 214268_s_at | MTMR4 | -1.978 |  | 0.3995 | < 0.0001 |  | 0.3361 | 0.0032 |
| 212277_at | MTMR4 | -1.978 |  | 0.3802 | 0.0002 |  | 0.3048 | 0.0078 |
| 221825_at | ANGEL2 | -1.968 |  | 0.3577 | 0.0005 |  | 0.5251 | < 0.0001 |
| 221826_at | ANGEL2 | -1.968 |  | 0.3205 | 0.002 |  | 0.3558 | 0.0017 |
| 225394_s_at | ZCRB1 | -1.876 |  | 0.3684 | 0.0003 |  | 0.5579 | < 0.0001 |
| 220140_s_at | SNX11 | -1.83 |  | 0.2231 | 0.0335 |  | 0.4179 | 0.0002 |
| 222598_s_at | NAV2 | 1.871 |  | -0.2077 | 0.0482 |  | -0.239 | 0.0389 |
| 222599_s_at | NAV2 | 1.871 |  | -0.3603 | 0.0005 |  | -0.2795 | 0.0152 |
| 218330_s_at | NAV2 | 1.871 |  | -0.3702 | 0.0003 |  | -0.3763 | 0.0009 |
| 224967_at | UGCG | 1.88 |  | -0.2271 | 0.0304 |  | -0.2888 | 0.012 |
| 201809_s_at | ENG | 1.889 |  | -0.2596 | 0.013 |  | -0.4188 | 0.0002 |
| 207610_s_at | EMR2 | 1.967 |  | -0.2966 | 0.0043 |  | -0.3657 | 0.0013 |
| 204642_at | S1PR1 | 2.126 |  | -0.2317 | 0.0271 |  | -0.5043 | < 0.0001 |
| **216979_at** | **NR4A3** | **2.19** |  | **-0.2508** | **0.0165** |  | **-0.255** | **0.0272** |
| 201744_s_at | LUM | 2.945 |  | -0.2651 | 0.0111 |  | -0.3356 | 0.0032 |

**Supplementary Table S1** The list of LINC00467-regulated genes whose expression was correlated with LINC00467 expression in HCC tissues analyzed by GEO dataset.
